# Underutilization of Statin Therapy for Secondary Prevention of Cardiovascular Disease Among Older Adults

**DOI:** 10.1089/pop.2018.0051

**Published:** 2019-02-01

**Authors:** Shirley Musich, Shaohung S. Wang, Kay Schwebke, Luke Slindee, Evonne Waters, Charlotte S. Yeh

**Affiliations:** ^1^ Research for Aging Populations, Optum, Ann Arbor, Michigan.; ^2^ Informatics & Data Science, Optum, Ann Arbor, Michigan.; ^3^ Medicare & Retirement, UnitedHealthcare Alliances, Minneapolis, Minnesota.; ^4^ AARP Services, Inc., Washington, District of Columbia.

**Keywords:** older adults, statin therapy, secondary CVD prevention

## Abstract

Secondary cardiovascular disease (CVD) clinical trials have demonstrated that higher intensity levels of statin therapy are more effective than lower levels in reducing mortality rates. Despite updated treatment guidelines, statin therapy may be underutilized, with evidence that females are treated less aggressively than males. The primary objective of this study was to determine the prevalence of statin utilization by varying therapy intensity by sex. The secondary objective was to document the benefits of statin therapy intensity levels on all-cause mortality for males and females. A 25% random sample of adults ≥65 years was utilized to identify those with established CVD. Inclusion criteria included: (1) 12-month pre period and (2) up to 30 months post period. Five categories of statin utilization were established: adherent to high-, moderate-, or low-intensity statin therapy, nonadherent, and no statins. Among eligible insureds (N = 49,530 males; N = 44,710 females), 20% of males and 12% of females were identified as high-intensity statin users. Mortality rates significantly increased similarly for males and females as statin therapy intensity decreased. Likewise, mortality hazard ratios indicated the most benefit from high-intensity statin therapy compared to all other categories. Statin therapy for secondary prevention of CVD is beneficial in reducing mortality for males and females but is underutilized, especially among females. Education programs among patients to increase heart health awareness and among physicians to promote the benefits of updated statin guidelines should be encouraged.

## Introduction

Statin therapy is a primary strategy for the secondary prevention of cardiovascular disease (CVD).^1–6^ The use of statins has been shown to significantly reduce the likelihood of repeat CVD hospitalizations and CVD-related mortality.^1–6^ Furthermore, numerous clinical trials and subsequent research have demonstrated that higher intensity levels of statin therapy are more effective than lower levels in reducing mortality rates.^7–16^ Despite this growing body of clinical evidence, statins tend to be underutilized, especially within selected subgroups including women and older adults.^4,10,11,13,15,17–22^

In 2013, the American College of Cardiology and American Heart Association (ACC/AHA) issued updated guidelines that represented a paradigm shift in the management of dyslipidemia and cardiovascular risk reduction among adults.^23^ Rather than treating to low-density lipoprotein cholesterol (LDL-C) targets, the new guidelines suggested that, at the time of CVD diagnosis, most adults aged 75 years and younger should instead receive high-intensity statin therapy regardless of their initial LDL-C values. Those older than 75 years of age should be evaluated individually but receive moderate-intensity statins at a minimum. The clinical trial evidence, however, has been most compelling for men aged 75 years and younger, with more limited information on women and adults aged 76 years and older.^7–9^ This lack of clarity may have contributed to the slow adoption of the updated guidelines by physicians, especially in community settings.^10,14^

The benefits of adding high-intensity statin therapy are most consistent for all-cause mortality with significant reductions in mortality rates within the short term (ie, 3 months) as well as longer term (ie, at least 10 years).^2,5,6,12,14–17,21,24^ The benefit of reduced nonfatal CVD-related hospitalizations is confounded by higher CVD risk levels associated with those on higher statin dosages.^12,14,15,25^ Offsetting the benefits of statins, there is a concern for undesirable side effects including musculoskeletal pain (eg, myalgia), muscle weakness, reduced mobility, and increased falls, especially associated with higher dosages or long-term statin use.^2,13,14,20,21,24,26–28^ Although side effects have been documented in 5%–10% of patients, the negative risks associated with statin therapy generally are associated with early stages of therapy.^2,13,24,29^

Disparities between the sexes in the treatment of dyslipidemia and secondary prevention of CVD have been well documented.^15,18–22^ Women consistently are less likely to have their lipids monitored and receive less aggressive drug treatment compared with men. Women also receive fewer and less timely CVD-related procedures compared to men.^19^ Thus, although statins are underutilized within older adult populations in general, the problem for women is even more pronounced.

No published research studies to date were found that considered the real-world adoption of ACC/AHA statin treatment guidelines among older adults with Medicare Supplement plans (ie, Medigap).^30^ In the United States, government-funded Medicare covers adults aged 65 years and older as well as those younger than age 65 with disabilities. Medicare fee-for-service plans (approximately 70% of all Medicare plans) pay about 80% of medical expenditures for these individuals but offer no prescription drug benefits. Although most (approximately 90%) of those with original fee-for-service Medicare purchase additional insurance plans to cover the remaining 20% of medical expenses, about 28% (currently approximately 10.2 million adults) have purchased Medigap coverage.^30^ Because this population may differ from the general older adult and/or overall Medicare populations, it was of interest to determine the implementation rates of the 2013 ACC/AHA updated statin treatment guidelines^23^ and to investigate the associated benefits of statin intensity levels on all-cause mortality rates by sex.

Thus, the primary objective was to determine the prevalence of statin utilization and intensity (high, moderate, or low) of treatment for secondary prevention of CVD among AARP Medicare Supplement insureds by sex. The secondary objective was to document the associated benefits of statin treatment on all-cause mortality by intensity level for males and females, controlling for other variables associated with mortality. This research was covered under the New England Institutional Review Board #120160532.

## Methods

### Study population

In 2015, approximately 4 million Medicare insureds were covered by an AARP^®^ Medicare Supplement plan insured by UnitedHealthcare Insurance Company. These plans are offered in all 50 states, Washington DC, and various US territories. A 25% random sample of 2015 AARP Medicare Supplement insureds with AARP^®^ MedicareRx (Part D) plans (approximately 55% of insureds) who were at least 65 years of age was utilized to identify patients with documented CVD suitable for secondary prevention strategies. The final study populations included 49,530 males and 44,710 females.

### Definition of CVD and statin therapy intensity levels

Patients with documented CVD were defined from *International Classification of Diseases, Ninth Revision* (ICD-9) or *International Classification of Diseases, Tenth Revision* (ICD-10) codes in the 12-month (2014) baseline period. A list of the codes utilized is available upon request. Patients must have had at least 1 CVD code on at least 2 different dates and have had at least 6 months and up to 30 months of follow-up coverage during 2015–2017.

Statin therapy intensity was classified according to the ACC/AHA cholesterol guidelines.^23^ Statin use was defined as receiving statin prescriptions during the 2015–2017 follow-up. A list of statins and relevant dosages are available upon request. Adherence was defined from the proportion of days covered (PDC) during the eligible follow-up months with “adherent” classified as having ≥70% of days covered. Patients must have remained on a consistent dosage during the 2.5 year follow-up period to be classified as high-intensity, moderate-intensity, or low-intensity statin users or as a nonadherent statin user <70% PDC regardless of statin level. Patients who down-titrated to lower statin levels (3%; n = 2912), up-titrated to higher levels (4%; n = 4678), or had mixed up and down titrations (2%; n = 2776) were excluded from the study population. In a subsequent analysis, the study team also considered levels of statin use in the baseline period.

### Covariates

Covariates were included to characterize individuals eligible for secondary prevention of CVD by sex and to adjust for other risk factors. These covariates included measures of demographics, socioeconomic factors, health status, and other characteristics taken from health plan eligibility and administrative medical claims.

Demographic questions included age and sex. Age groups were defined as: 64–69; 70–74; 75–79; 80–84; and ≥85 years. Geographic regions (Northeast, South, Midwest, and West); low (less than 15% nonwhite), medium (15% to 59% nonwhite), and high (≥60% nonwhite) minority areas; and low (<$40,179), medium ($40,179 to <$ 57,199), and high (≥$57,199) median household income levels were geocoded from zip codes. AARP Medicare Supplement plan types were grouped by cost-sharing levels, including high-level coverage plans with minimal co-payments or deductibles, less comprehensive medium-level coverage, and all other plans. Two measures of health services access were calculated as acute hospital beds per 100,000 capita and primary care physicians per 100,000 capita.

### Prevalence of common chronic conditions

Four chronic conditions (depression, hyperlipidemia, hypertension, and obesity/overweight) identified using Evidence-Based Medicine (Symmetry EBM Connect^®^ Version 8.3; Optum, Eden Prairie, MN, USA) software were included in these analyses. This software was developed to measure quality of care from health care claims data using a defined set of measures for evidence-based care associated with various medical conditions. Cardiovascular risk factors were identified from the aforementioned chronic conditions.

Eleven chronic conditions were defined from Charlson Comorbidity Index (CCI) diagnoses codes: AIDS/HIV, cancer, chronic obstructive pulmonary disease (COPD), dementia, diabetes, heart problems, liver disease, peptic ulcer disease, renal disease, rheumatoid arthritis, and stroke. CCI is a measure of the risk of 1-year all-cause mortality attributable to selected comorbidities that also has been shown to be highly predictive of morbidity and health care expenditures.^31^

### Injurious falls/hip fractures and musculoskeletal pain

Injurious falls requiring medical services or hip fractures, as a combined measure, were defined from suggested Healthcare Effectiveness Data and Information Set diagnoses codes.^32^ Falls or hip fractures have been associated with the use of statins. Falls or hip fractures were documented from these selected diagnoses codes at any time during the baseline period (2014).

Musculoskeletal pain often associated with statin use was identified from ICD-9 or ICD-10 diagnosis codes. Musculoskeletal pain in the baseline period (2014) was used as a control variable in subsequent regression models.

### Outcomes: mortality and CVD hospitalization

Mortality was determined from the AARP Medicare Supplement eligibility files maintained by UnitedHealthcare. Patients must have had a 12-month baseline and at least 6 months of plan eligibility in the follow-up and must have been alive through June 2015, but then were followed until death or the end of the follow-up in June 2017.

CVD hospitalization patients were identified from ICD-9 or ICD-10 codes used for the original study population identification. Patients were followed until a CVD hospitalization or until the end of the follow-up in June 2017.

### Statistical models

Demographic variables for males and females were statistically tested across the 5 statin use categories using chi-square for categorical variables or *t* tests for continuous variables considering *P* < 0.05 as significant. All analyses were completed using SAS Enterprise Guide Version 7.1 (SAS Institute Inc., Cary, NC, USA). Missing demographic variables (income, minority, and location) were treated as separate categories, although because these variables were calculated from address zip codes, missing data were minimal (<1.5% for any 1 variable). Consequently no imputation of data was utilized.

Unadjusted mortality and CVD hospitalization rates by statin use categories were determined by sex. Propensity score methodology was utilized to weight for the likelihood to receive high-intensity statins using variables listed in Table 1.^33,34^ Results were subsequently regression adjusted to control for any significant variable differences that may have remained after the propensity weighting.

**Table 1.** Unadjusted Characteristics Associated with Statin Use Categories: Males

	*Study sample*	*Statin users*				*Statin nonusers*	
*Characteristics*	*Total % or mean*	*High intensity % or mean*	*Moderate intensity % or mean*	*Low intensity % or mean*	*Nonadherent % or mean*	*No statin % or mean*	P *value*
Number	49,530	9885	16,005	2045	12,878	8717	
Age, years	76.1	74.0	76.1	77.5	76.1	78.1	<0.0001
65–69	20.2	26.7	18.8	14.1	20.2	16.8	<0.0001
70–74	26.5	31.7	27.2	24.3	26.0	20.7	
75–79	22.3	22.3	23.1	23.3	22.6	20.1	
80–84	16.3	12.8	16.8	19.8	16.4	18.5	
≥85	14.7	6.6	14.2	18.6	14.9	24.0	
Minority (from zip code)							
Low	50.7	50.9	50.5	52.6	50.0	51.6	0.002
Medium	44.9	45.4	45.2	43.0	45.3	43.5	
High	2.9	2.4	2.9	3.0	3.2	3.3	
Income (from zip code)							
Low	14.9	12.3	14.5	15.8	15.7	17.3	<0.0001
Medium	34.5	33.6	34.5	35.0	34.9	35.1	
High	50.3	53.9	50.9	49.0	49.1	47.2	
Region							
Midwest	15.7	15.9	16.4	15.1	15.3	15.3	<0.0001
Northeast	26.2	25.3	26.3	26.1	25.7	27.6	
South	40.9	39.4	40.9	41.9	41.5	41.3	
West	16.9	19.2	16.2	16.8	17.1	15.4	
Health care supply							
Acute hospital beds per 100,000	231.4	226.5	231.8	233.1	231.3	236.1	<0.0001
PCP per 100,000	131.1	133.4	130.9	131.5	130.5	129.9	<0.0001
Plan type							
High coverage	80.3	81.8	80.5	80.0	80.3	78.5	<0.0001
Medium coverage	2.6	2.2	2.6	3.0	2.7	2.6	
Other	17.1	16.0	17.0	17.0	17.0	18.9	
CCI conditions							
AIDS/HIV	0.1	0.1	0.1	0.1	0.1	0.1	0.84
COPD	28.3	24.0	26.4	30.6	29.5	34.2	<0.0001
Cancer	20.9	17.1	20.1	22.0	22.1	24.6	<0.0001
Dementia	3.9	1.7	3.0	3.8	4.3	7.3	<0.0001
Diabetes	38.1	39.3	38.0	38.8	40.8	32.9	<0.0001
Heart problems	58.0	54.9	55.2	57.5	59.2	64.7	<0.0001
Liver disease	4.6	3.3	3.8	4.7	5.2	6.5	<0.0001
Peptic ulcer disease	1.9	1.4	1.7	2.1	2.1	2.3	<0.0001
Renal disease	20.1	18.1	18.6	23.8	21.6	21.9	<0.0001
Rheumatoid arthritis	3.1	2.5	2.8	3.6	3.2	4.1	<0.0001
Stroke	34.0	31.9	33.6	35.8	35.6	34.4	<0.0001
EBM conditions							
Depression	3.9	3.4	3.5	3.8	4.3	4.8	<0.0001
Hyperlipidemia	83.4	92.7	90.5	89.1	86.4	54.3	<0.0001
Hypertension	91.5	92.2	91.6	93.4	92.1	89.4	<0.0001
Obesity/overweight	13.3	14.7	12.8	13.7	13.8	12.1	<0.0001
Fall/hip fracture	5.1	3.1	4.5	6.2	5.5	7.6	<0.0001
Musculoskeletal pain	4.1	3.2	3.4	3.6	4.7	5.6	<0.0001
# of risk factors	2.3	2.4	2.3	2.3	2.3	2.0	<0.0001

Risk factors include diabetes, hypertension, hyperlipidemia, and obesity from diagnosis codes. Missing for income, minority, and region were calculated separately but not shown in the table for brevity.

CCI, Charlson Comorbidity Index; COPD, chronic obstructive pulmonary disease; EBM, Evidence-Based Medicine; PCP, primary care physicians.

Cox proportional hazard regression analyses weighted by the inverse propensity score were used to determine the association of high-intensity statins relative to the other statin use groups with all-cause mortality and CVD hospitalizations using all of the demographic, socioeconomic, and health status variables in Table 1. Adjusted hazard ratios were calculated by sex comparing high-intensity statin utilization risk of mortality or CVD hospitalizations to other statin categories. Subsequently, each category of statin utilization was compared to each other level to establish statistical differences between categories.

## Results

Overall, among the random study sample with Part D prescription drug plans (n = 548,429), 85% (n = 412,093) met eligibility criteria for age of at least 65 years and continuous plan enrollment from January 2014 through June 2017 or as long as each patient could be followed. Of these, 5% (n = 24,967) were excluded because of statin switching patterns. Of the remaining patients (n = 387,126), 20% (n = 100,800) were identified as established CVD patients. After removing non-statin users who had used statins in the baseline period, the final study populations included 94,240 patients (49,530 males and 44,710 females) (Tables 1 and 2). Male CVD patients (53%) were more likely to be 70–74 years (27%), white (50%), and living in the South (41%). Female CVD patients (47%) were more likely to be ≥85 years (27%), white (49%), and living in the South (39%). On average, female CVD patients were 3 years older than males (79 vs. 76 years). Reflecting this age difference, females were more likely to suffer from COPD, dementia, rheumatoid arthritis, stroke, and to have had a fall/hip fracture compared to males.

**Table 2.** Unadjusted Characteristics Associated with Statin Use Categories: Females

	*Study sample*	*Statin users*				*Statin nonusers*	
*Characteristics*	*Total % or mean*	*High intensity % or mean*	*Moderate intensity % or mean*	*Low intensity % or mean*	*Nonadherent % or mean*	*No statin % or mean*	P *value*
Number	44,710	5268	12,364	2017	12,037	13,024	
Age, years	78.7	75.9	78.3	79.5	78.1	80.8	<0.0001
65–69	14.8	22.1	14.2	12.2	16.2	11.7	<0.0001
70–74	20.0	25.3	21.3	18.3	21.1	15.6	
75–79	19.1	21.3	20.6	18.3	19.7	16.4	
80–84	19.1	17.6	19.9	22.3	19.0	18.6	
≥85	27.0	13.7	24.1	28.8	24.0	37.7	
Minority (from zip code)							
Low	48.5	48.8	48.5	50.1	47.6	48.9	0.01
Medium	46.6	46.8	46.8	45.3	46.8	46.5	
High	3.7	3.3	3.6	3.5	4.2	3.4	
Income (from zip code)							
Low	15.7	13.9	15.4	15.1	16.5	16.0	<0.0001
Medium	35.1	34.7	35.9	37.1	34.9	34.4	
High	48.9	51.1	48.4	47.6	48.1	49.3	
Region							
Midwest	16.5	17.3	17.1	17.7	15.4	16.4	<0.0001
Northeast	29.7	28.3	30.2	28.5	29.6	30.2	
South	39.0	38.7	38.5	38.3	40.3	38.4	
West	14.5	15.6	13.8	15.3	14.2	14.7	
Health care supply							
Acute hospital beds per 100,000	235.2	228.8	235.9	236.58	235.5	236.5	<0.0001
PCP per 100,000	131.4	133.4	131.4	131.4	130.9	131.0	0.001
Plan type							
High coverage	75.1	77.5	75.8	74.4	76.0	72.9	<0.0001
Medium coverage	2.8	2.9	2.7	3.2	2.9	2.8	
Other	22.0	19.6	21.5	22.4	21.1	24.3	
CCI Conditions							
AIDS/HIV	0.0	0.1	0.0	0.0	0.0	0.0	0.05
Cancer	14.6	13.0	14.4	14.8	14.6	15.5	0.001
COPD	33.8	30.4	32.1	34.0	35.9	35.0	<0.0001
Dementia	7.1	3.3	5.3	5.8	6.5	11.2	<0.0001
Diabetes	31.1	38.7	33.8	29.0	34.9	22.3	<0.0001
Heart problems	62.1	57.2	59.6	59.3	61.2	67.6	<0.0001
Liver disease	4.7	3.4	4.0	4.6	5.1	5.4	<0.0001
Peptic ulcer disease	2.2	2.3	2.0	1.6	2.4	2.2	0.15
Renal disease	17.1	18.1	17.2	16.7	18.6	15.2	<0.0001
Rheumatoid arthritis	6.9	5.6	5.9	6.1	7.3	8.2	<0.0001
Stroke	40.6	41.2	42.0	40.6	42.1	37.8	<0.0001
EBM Conditions							
Depression	8.4	7.5	7.9	6.8	8.6	9.3	<0.0001
Hyperlipidemia	75.5	93.1	90.5	89.3	84.8	43.5	<0.0001
Hypertension	92.3	94.6	93.9	93.4	93.6	88.5	<0.0001
Obesity/Overweight	13.2	15.7	14.0	12.4	14.3	10.5	<0.0001
Fall/hip fracture	10.2	7.4	8.8	9.0	10.2	12.8	<0.0001
Musculoskeletal pain	7.2	5.7	5.8	7.5	8.5	7.8	<0.0001
# of risk factors	2.1	2.4	2.3	2.2	2.3	1.7	<0.0001

Risk factors include diabetes, hypertension, hyperlipidemia, and obesity from diagnosis codes. Missing for income, minority, and region were calculated separately but not shown in the table for brevity.

CCI, Charlson Comorbidity Index; COPD, chronic obstructive pulmonary disease; EBM, Evidence-Based Medicine; PCP, primary care physicians.

Overall, among male CVD patients, 20% used high-intensity statins with 32% using moderate intensity, 4% low intensity, 26% nonadherent, and 18% receiving no statins. Among female CVD patients, 12% used high-intensity statins with 28% using moderate intensity, 5% low intensity, 27% nonadherent, and 29% receiving no statins. Among both male and female statin users, statin intensity levels in the follow-up period were generally consistent with levels established in the baseline period: 92% of high-intensity, 91% of moderate-intensity, and 84% of low-intensity users continued statin prescriptions after their CVD diagnosis at the same level as the baseline period (data not shown) (Tables 1 and 2).

**FIG. 1.**
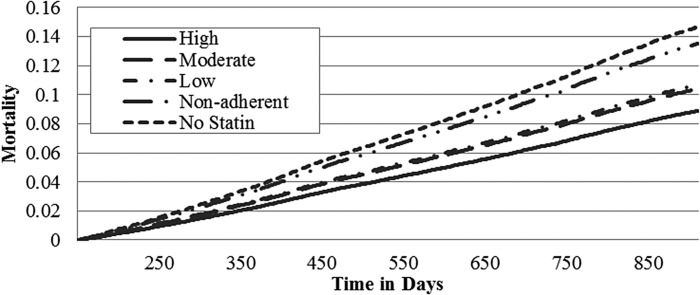
Adjusted mortality curves for different intensities of statin therapy – male. Curves are adjusted for propensity to receive a high-intensity statin and regression adjusted with variables from Table 1. For males, all survival hazard ratios are significantly different (*P* < 0.001) except for moderate and low nonsignificant (*P* > 0.50).

**FIG. 2.**
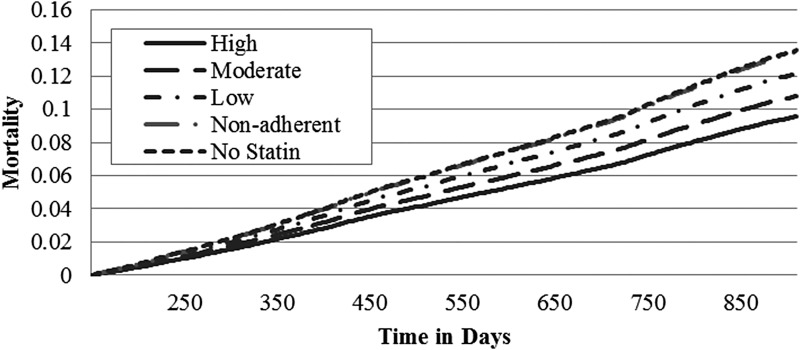
Adjusted mortality curves for different intensities of statin therapy – female. Curves are adjusted for propensity to receive a high-intensity statin and regression adjusted with variables from Table 1. For females, all survival hazard ratios are significantly different (*P* < 0.001) except for moderate vs. low; low vs. nonadherent or no statins; and nonadherent vs. no statins (*P* > 0.07).

Adjusted mortality rates increased from 8.9% to 14.7% for males and from 9.6% to 13.5% for females in a dose-related relationship as the statin intensity decreased from high-intensity statin use to no statins (Table 3). For males, the adjusted mortality hazard ratios demonstrated that any statin use, including being nonadherent, was better than no statins. For females, only high- and moderate-intensity statins significantly reduced mortality rates/hazard ratios. Males using no statins had a 65% increased mortality rate compared to high-intensity statin users; females using no statins had a 41% increased mortality rate compared to high-intensity users. For males, each of the adjusted mortality rates and hazard ratios was significantly different from all other categories with the exception of moderate- and low-intensity statins, which were not different. For females, only high- and moderate-intensity statin users had significantly reduced mortality rates compared to low-intensity, nonadherent, or no statins (Figs. 1 and 2; Table 3).

**Table 3.** Outcomes for Male and Female High-Intensity Statins Versus Moderate- and Low-Intensity, Nonadherent, and No Statins

	*Statin users*				
*Outcome measures*	*High intensity*	*Moderate intensity*	*Low intensity*	*Nonadherent*	*No statins*
**Male**					
Number	9885	16,005	2045	12,878	8717
Unadjusted mortality rates (%)	7.9	11.4	14.3	16.2	22.8
Adjusted mortality rates (%)^*^	8.9	10.4	10.7	13.5	14.7
					
Unadjusted CVD hospitalization rates (%)	29.9	28.2	28.3	35.9	26.3
Adjusted CVD hospitalization rates (%)	38.9	33.9	31.7	45.1	32.6
					
Survival, hazard ratio (95% CI)^*^	Ref.	1.17 (1.08–1.27)	1.20 (1.05–1.38)	1.52 (1.40–1.65)	1.65 (1.52–1.79)
Repeat CVD hospitalization, hazard ratio (95% CI)^*^	Ref.	0.87 (0.83–0.92)	0.82 (0.75–0.89)	1.16 (1.00–1.22)	0.84 (0.79–0.88)
Female					
Number	5268	12,364	2017	12,037	13,024
Unadjusted mortality rates (%)	8.4	11.3	14.2	15.3	20.6
Adjusted mortality rates (%)^*^	9.6	10.8	12.1	13.4	13.5
					
Unadjusted CVD hospitalization rates (%)	29.0	24.4	23.0	31.4	18.7
Adjusted CVD hospitalization rates (%)^*^	35.8	28.4	26.0	38.2	23.7
					
Survival, hazard ratio (95% CI)^*^	Ref.	1.13 (1.04–1.23)	1.27 (1.11–1.46)	1.41 (1.29–1.53)	1.42 (1.30–1.55)
Repeat CVD hospitalization, hazard ratio (95% CI)^*^	Ref.	0.80 (0.75–0.84)	0.73 (0.65–0.81)	1.07 (1.01–1.13)	0.65 (0.62–0.71)

^*^ All results adjusted for propensity to be prescribed a high-intensity statin and regression adjusted for variables in Table 1. For males, all survival hazard ratios significantly different (*P* < 0.001) except for moderate and low nonsignificant (*P* > 0.50). All CVD hospitalizations significantly different (*P* < 0.001) except for moderate vs. low or no statins and low vs. no statins (*P* > 0.08). For females, all survival hazard ratios significantly different (*P* < 0.001) except for moderate vs. low; low vs. nonadherent or no statins; and nonadherent vs. no statins (*P* > 0.07). All CVD hospitalizations significantly different (*P* < 0.001) except for moderate vs. low and low vs. no statins (*P* > 0.07).

CVD, cardiovascular disease; CI, confidence interval.

CVD hospitalizations demonstrated a different pattern. Consistent with increasing cardiovascular risk factors associated with increased statin intensity, the dose-related relationship was in the opposite direction, with higher CVD hospitalizations associated with high-intensity statins, and fewer hospitalizations among those with lesser or no statin use for both males and females (Tables 1 and 2). The highest rate of CVD hospitalizations was associated with the nonadherent category (Table 3).

## Discussion

In this population of AARP Medicare Supplement insureds, about 20% were identified as a population suitable for secondary prevention of CVD. Among males with CVD, 20% utilized high-intensity statins compared to 12% for females with CVD; 32% of males utilized moderate-intensity statins compared to 28% of females. Thus, considering the updated 2013 ACC/AHA guidelines recommending either high- or moderate-intensity statins for all older adults who can tolerate the drugs, statins were underutilized in this real-world Medicare Supplement population, but for women the problem was more acute. This distribution of statin utilization by intensity level is generally consistent with studies prior to 2013, reflecting older guidelines recommending treatment to LDL-C levels.^4,5,10–15,26,27^ Although teaching hospitals and Veterans Affairs have published evidence of phasing in the updated guidelines to secondary CVD treatment protocols since 2013,^10,14^ community physicians seem to have been less likely to adopt the newer approaches.

The all-cause mortality benefit, adjusted for demographic, socioeconomic, and health status variables associated with mortality, was evident in a dose-related response relationship rate with mortality increasing as statin intensity decreased for both males and females. Adjusted hazard ratios indicated a 65% reduction for males and a 42% reduction for females in the risk of mortality associated with high-intensity statins compared to no statin utilization. These results are consistent with other studies demonstrating that the mortality benefits associated with statins were similar for men and women.^15,18,21,35^ For men in the present study, however, any statin utilization was significantly better than none. For women, only high- and moderate-intensity statins significantly reduced mortality. These results reinforce the recommendation for more CVD studies conducted on women separately from men, including assessment of cardiovascular risk factors, specific statins, and related dosage impacts.^10,18,35^

In contrast, the pattern for CVD hospitalizations across the statin use categories suggests community physicians prescribed statin levels based on cardiovascular risk status.^2,6,11–13,15,16,29^ Consequently, the “dose”-related relationship was reversed with those on high statins more likely to experience a CVD event than those on lower levels or no statins. That higher dosages of statins for both males and females are typically associated with higher CVD risks is consistent in the statin literature.^2,6,11–13,15,16,29^ One longitudinal research study documented that 5–10 years of statin therapy may be required to reduce the risk of nonfatal repeat events for high-intensity utilizers to no statin use levels.^15^ Thus, once statin therapy is initiated, high adherence over long periods of time is required to maintain the designated benefits. As confirmation, nonadherent males and females in the present study results had mortality rates similar to those receiving no statins.

Overall, nonadherence rates were 26% for males and 27% for females, lower than levels documented in other research studies.^26,27,29,36,37^ This may have been a reflection of the study population that focused on sustained statin therapy at a given level over the 2.5-year follow-up period. Any issues with side effects may already have been addressed.^2,13,24,29^ As in other studies, no evidence was found of an impact from side effects of statins associated with dosage levels^2,20,21,24,27,28^; however, more detailed information on reasons for nonadherence was not available. About 18% of male and 29% of female CVD patients received no statins. Nevertheless, this percentage range for receiving no statins is consistent with the statin literature^4,5,10–13,15,16^ and, while those not taking statins may include those unable to tolerate the drug, other reasons for not taking or refusing treatment include already low levels of LDL-C, not being convinced of the benefits of treatment, polypharmacy, or possible cost issues.^4,10,11,13^

As anticipated, disparities between the sexes in this study were consistently evident among overall females and among females in various demographic categories, such as age groups, regions of the country, and income levels (data not shown). Although the reduction in mortality rates for females given statin treatment was consistent with the benefit realized among males, females consistently continue to be treated at lower rates. The problem of undertreatment of women compared to men, however, is persistent in the scientific literature.^15,18–22^ The problem, while well identified, is multifaceted, involving both physicians and patients and, consequently, defies an easy solution. Suggestions for better physician–patient communication with more extensive follow-up, especially in the early months of starting statin therapy, may be beneficial.^36,37^ The perception of risk associated with CVD may be underestimated for women by both physicians and patients, hence continuing education remains critical.^10^ Continued focus on the benefits of the updated statin guidelines and treatment protocols for community-based physicians also may be warranted. To further promote physician compliance with statin guidelines,^38,39^ health plans and clinic organizations also might offer information on the benefits of statins to patients to empower patient–physician discussions, teaching sessions for physicians to build awareness of updated guidelines, general communications on guideline benefits, and/or reimbursement schemes tied to statin use quality measures.

As a note, newer clinical guidelines for managing dyslipidemia were issued during the time of this study: by the European Society of Cardiology/European Atherosclerotic Society in 2016 and by the American Association of Clinical Endocrinologists and American College of Endocrinology in 2017.^40^ These guidelines were an attempt to update clinicians on the latest developments in lipid management. Numerous recommendations on a broad range of clinical scenarios were included, notably with a renewed focus on LDL-C levels. The guidelines acknowledged that lower LDL-C levels than previously targeted (eg, <55 mg/dL) may be desirable for selected patients at high risk for CVD events. Although LDL-C remained the primary target, additional recommendations were added to include triglycerides and high-density lipoproteins in a more comprehensive approach to lipid management.^40^

This study has some limitations. The study population of AARP Medicare Supplement insureds may not generalize to all older adults or to those with other Medicare or Medicare Supplement plans. Prescription medication use was defined from administrative pharmacy databases reflecting purchase patterns but not necessarily consumption patterns. Mortality records did not include cause of death; hence, specific causes of death could not be determined. More information on community physician attitudes would be helpful in encouraging the adoption of recommended ACC/AHA guidelines. Strengths of the study include a large real-world community-based study population that included a representative male/female distribution.

## Conclusion

Overall, in this population of Medicare Supplement insureds, statin therapy was underutilized. Thus, many older adults are not receiving the additional mortality benefits of statin treatment, especially benefits associated with high-intensity statins. Disparities between the sexes were evident with women less likely to receive statins at any level compared to men. Community physicians appear to predominately treat to cardiovascular risk levels rather than to the new guidelines for secondary prevention of CVD. Continuing education to increase physician awareness of the benefits of revised guidelines and updated treatment protocols should be encouraged.
